# Complete Genome Sequence of Bifidobacterium scardovii Strain JCM 12489^T^, Isolated from Human Blood

**DOI:** 10.1128/genomeA.00285-15

**Published:** 2015-04-09

**Authors:** Hidehiro Toh, Kenshiro Oshima, Akiyo Nakano, Naoko Yamashita, Erica Iioka, Rina Kurokawa, Hidetoshi Morita, Masahira Hattori

**Affiliations:** a Medical Institute of Bioregulation, Kyushu University, Fukuoka, Japan; b Graduate School of Frontier Sciences, University of Tokyo, Kashiwa, Japan; c Department of Microbiology and Immunology, Teikyo University School of Medicine, Tokyo, Japan; d School of Veterinary Medicine, Azabu University, Sagamihara, Japan

## Abstract

Bifidobacterium scardovii strain JCM 12489^T^ was isolated from human blood and has the largest bifidobacterial genome reported to date. Here, we report the complete genome sequence of this organism. This paper is the first report demonstrating the fully sequenced and completely annotated genome of a B. scardovii strain.

## GENOME ANNOUNCEMENT

Although Bifidobacterium species are frequently isolated from the human intestine, B. scardovii strain JCM 12489^T^ (DSM 13734^T^) was isolated from human blood (1). B. scardovii was also isolated from a patient with a genitourinary tract wound infection (2), and recurrent urinary infection with B. scardovii has been reported (3). B. scardovii forms a distinct branch in the phylogenetic tree of the genus Bifidobacterium (4).

The genome sequence of JCM strain 12489^T^ was determined by a whole-genome shotgun strategy using Sanger sequencing (3730xl DNA sequencers) and 454 pyrosequencing (GS-FLX sequencers). We constructed small-insert (2-kb) and large-insert (10-kb) genomic DNA libraries and generated 38,400 (9.0-fold, 3730xl) and 373,749 reads (28.8-fold, GS-FLX) from the B. scardovii JCM 12489^T^ genome. The 454 pyrosequencing reads were assembled using the Newbler assembler software. A hybrid assembly of 454 and Sanger reads was performed using the Phred-Phrap-Consed program. Gap closing and resequencing of low-quality regions were conducted by Sanger sequencing to obtain the high-quality finished sequence. The overall accuracy of the finished sequence was estimated to have an error rate of <1 per 10,000 bases (Phrap score of ≥40). An initial set of predicted protein-coding genes was identified using Glimmer version 3.0 (5). Genes consisting of <120 bp and those containing overlaps were eliminated. The tRNA genes were predicted by tRNAscan-SE (6), and the rRNA genes were detected by a BLASTn search using known Bifidobacterium rRNA sequences as queries.

The genome sequence of B. scardovii JCM 12489^T^ consists of a circular chromosome of 3,158,347 bp with no plasmid. This is the largest bifidobacterial genome reported to date and is 326 kb larger than that (2.8 Mb) of Bifidobacterium longum subsp. infantis strain JCM 1222^T^ (ATCC 15697^T^), whose genome was previously the largest in the Bifidobacterium species (7, 8). The genome of JCM 12489^T^ has the highest GC content (64.6%) in the Bifidobacterium species to date. JCM 12489^T^ contained a clustered regularly interspaced short palindromic repeats (CRISPR) (9) region (2,307,006 to 2,308,804), and seven CRISPR-associated genes (BBSC_1927 to BBSC_1933) were encoded upstream of the CRISPR region. The chromosome contains 2,572 predicted protein-coding genes. Using the CAZy database (10), 95 genes for glycosyl hydrolase (27 families) were found in the genome, indicating that JCM 12489^T^ contains a larger number of genes for glycosyl hydrolase in the Bifidobacterium species. Of the 95 glycosyl hydrolase genes, only one gene (BBSC_0167) contained the LPXTG motif, which is required for linking to the cell wall envelope, whereas Bifidobacterium bifidum JCM 1255^T^ contained 13 glycosyl hydrolase genes with the LPXTG motif (accession no. AP012323). The genome information of this species will be useful for further studies of its physiology, taxonomy, ecology, and clinical aspects.

### Nucleotide sequence accession number.

The sequence data for the genome have been deposited in DDBJ/GenBank/EMBL under the accession number AP012331.
